# Local Ancestry Inference in a Large US-Based Hispanic/Latino Study: Hispanic Community Health Study/Study of Latinos (HCHS/SOL)

**DOI:** 10.1534/g3.116.028779

**Published:** 2016-03-22

**Authors:** Sharon R. Browning, Kelsey Grinde, Anna Plantinga, Stephanie M. Gogarten, Adrienne M. Stilp, Robert C. Kaplan, M. Larissa Avilés-Santa, Brian L. Browning, Cathy C. Laurie

**Affiliations:** * Department of Biostatistics, University of Washington, Seattle, Washington 98195; † Department of Epidemiology and Population Health, Albert Einstein College of Medicine, Bronx, New York 10461; ‡ Division of Cardiovascular Sciences, National Heart, Lung, and Blood Institute, National Institutes of Health, Bethesda, Maryland 20892; § Division of Medical Genetics, Department of Medicine, University of Washington, Seattle, Washington 98195

**Keywords:** Hispanic/Latino, local ancestry, principal components analysis

## Abstract

We estimated local ancestry on the autosomes and X chromosome in a large US-based study of 12,793 Hispanic/Latino individuals using the RFMix method, and we compared different reference panels and approaches to local ancestry estimation on the X chromosome by means of Mendelian inconsistency rates as a proxy for accuracy. We developed a novel and straightforward approach to performing ancestry-specific PCA after finding artifactual behavior in the results from an existing approach. Using the ancestry-specific PCA, we found significant population structure within African, European, and Amerindian ancestries in the Hispanic/Latino individuals in our study. In the African ancestral component of the admixed individuals, individuals whose grandparents were from Central America clustered separately from individuals whose grandparents were from the Caribbean, and also from reference Yoruba and Mandenka West African individuals. In the European component, individuals whose grandparents were from Puerto Rico diverged partially from other background groups. In the Amerindian ancestral component, individuals clustered into multiple different groups depending on the grandparental country of origin. Therefore, local ancestry estimation provides further insight into the complex genetic structure of US Hispanic/Latino populations, which must be properly accounted for in genotype-phenotype association studies. It also provides a basis for admixture mapping and ancestry-specific allele frequency estimation, which are useful in the identification of risk factors for disease.

Hispanic Americans are a significant population group in the US, and comprised 16% of the US population in 2010. Due to the colonial history of the Americas, Hispanics derive their ancestry mostly from European, African, and indigenous American (Amerindian) ancestors, with many Hispanic/Latinos having admixed genomes that are mosaics of ancestry from two or more of these continental sources. Statistical methods have been developed to infer the local ancestry at each point of the genome (Price *et al.* 2009; Maples *et al.* 2013). The local ancestry at a genomic position describes the continental origin of the individual’s two chromosomes (one from each parent) at that position. For example, each of the two alleles at each variant site in the genome of a Hispanic/Latino individual can be designated as deriving from African, European, or Amerindian ancestors through local ancestry estimation.

Inferred local ancestry can be used for genetic association analysis via admixture mapping, in which one tests for association between the number of copies of an ancestry at a genomic location and the trait of interest (Patterson *et al.* 2004). Admixture mapping can be more powerful than SNP association mapping when the causal SNP is not genotyped and is difficult to impute, but has very different frequencies between continental populations (Hoggart *et al.* 2004). Inferred local ancestry can also be used to help interpret results from SNP association mapping, as one can infer the continental origin, or continent-specific allele frequencies, of variants that are significantly associated with the trait of interest (Gravel *et al.* 2013). An additional application of inferred local ancestry is to better understand the underlying within-continental structure in admixed populations (Moreno-Estrada *et al.* 2013), which can be used to inform decisions about association analysis, such as the use of population grouping variables.

Previous studies have analyzed within-ancestry population structure using local ancestry of individuals from Mexico, Central and South America, and the Caribbean islands. Moreno-Estrada *et al.* (2013) analyzed local ancestry of 330 individuals from the Caribbean basin (individuals from Cuba, Puerto Rico, Hispaniola, Honduras, and Colombia, and individuals from the Yukpa, Bari, and Warao Amerindian populations). Gravel *et al.* (2013) analyzed local ancestry of individuals from the 1000 Genomes Colombian, Mexican-American, and Puerto-Rican populations. Moreno-Estrada *et al.* (2014) analyzed local ancestry in over 1000 Mexicans. Homburger *et al.* (2015) analyzed local ancestry of 437 individuals from five South American countries (Colombia, Ecuador, Peru, Chile, and Argentina).

A previous large study of Latinos in the US, derived from the 23andMe database, calculated local ancestry for over 8000 Latinos (Bryc *et al.* 2015). The local ancestry estimates were used to calculate global ancestry proportions and to study ancestry tract lengths, but were not used to investigate within-ancestry population structure.

The study reported here differs from the previous studies of admixed Hispanics/Latinos in that it focuses on within-ancestry structure of individuals living in the US and is much larger than previous studies. It includes genetic data on over 12,000 self-identified Hispanic/Latino individuals, living in four major cities across the US, who are participants in the Hispanic Community Health Study/Study of Latinos (HCHS/SOL). We previously analyzed the ‘global’ ancestry of individuals in this cohort, estimating overall proportions of continental ancestries and detecting variation among six self-identified background groups (Cuban, Dominican, Puerto Rican, Mexican, Central, and South American) (Conomos *et al.* 2016). Here, we extend these analyses by utilizing local ancestry estimates to investigate within-continental ancestries of Hispanic/Latino individuals in the US. We also provide evaluation of methodological issues in local ancestry estimation, such as the selection of reference individuals, treatment of the X chromosome, and ancestry-specific principal components analysis (PCA).

## Materials and Methods

The HCHS/SOL (http://www.cscc.unc.edu/hchs/; Lavange *et al.* 2010; Sorlie *et al.* 2010) is a community-based cohort study of 16,415 self-identified Hispanic/Latino persons aged 18–74 years old, from randomly selected households in four US field centers (Chicago, IL; Miami, FL; Bronx, NY; San Diego, CA) with baseline examination (2008–2011) and yearly telephone follow-up assessment for at least 3 yr. The HCHS/SOL cohort includes participants who self-identified as having Hispanic/Latino background, the largest groups being Central American (n = 1730), Cuban (n = 2348), Dominican (n = 1460), Mexican (n = 6471), Puerto-Rican (n = 2728), and South American (n = 1068). The goals of the HCHS/SOL are to describe the prevalence of risk and protective factors for chronic conditions (*e.g.*, cardiovascular disease (CVD), diabetes, and pulmonary disease), and to quantify all-cause mortality, fatal and nonfatal CVD and pulmonary disease, and pulmonary disease exacerbation over time. The HCHS/SOL study was approved by institutional review boards at participating institutions, and written informed consent was obtained from all participants. The individuals were genotyped on an Illumina Omni 2.5M array with additional custom content, and the genotype and phenotype data are posted on dbGaP (accession numbers phs000880.v1.p1 and phs000810.v1.p1). We chose not to investigate Asian ancestry in this study, because the overall proportion of Asian ancestry in the sample is very low. After quality control and removal of 19 outlier individuals with significant Asian ancestry (Conomos *et al.* 2016), our analyses included 12,774 individuals who consented to genetic studies.

Inference of local ancestry requires the use of reference panels representing the ancestral populations. We considered two reference panels. The first was derived from the Human Genome Diversity Project (HGDP) (Li *et al.* 2008) and The 1000 Genomes Project Consortium (2012) (henceforth referred to as the HGDP reference). The second was derived from data published in Reich *et al.* (2012) combined with data from the 1000 Genomes Project (henceforth referred to as the Reich2012 reference).

For the HGDP reference, we first selected individuals from populations in the HGDP and 1000 Genomes data with European, West African, Amerindian, or East Asian ancestry. These collections are broadly consented for research and publicly available. We ran an unsupervised ADMIXTURE analysis with K = 4 populations (Alexander *et al.* 2009), and retained individuals with at least 90% estimated ancestry from one of the inferred ancestral populations, excluding East Asia. This resulted in 195 West Africans (from Nigeria, Senegal, and Barbados), 63 Amerindians (from Peru, Mexico, Ecuador, Brazil, and Colombia), and 527 Europeans (from Spain, Italy, the British Isles, France, and Utah). The HGDP individuals were genotyped using the Illumina HumanHap650Y array, and the 1000 Genomes individuals were genotyped on the Illumina Omni2.5M array. After intersection with the HCHS/SOL SNPs, 419,645 SNPs remained.

The Reich2012 reference included samples from HGDP, HapMap3, 1000 Genomes, and additional Amerindians. We included only individuals consented for both population genetics and health-related research. The genetic data were processed as for the HGDP reference, resulting in 198 West Africans, 154 Amerindians, and 516 Europeans. This reference set has a larger number of Amerindian individuals than the HGDP reference, but a smaller number of SNPs (236,736) after intersection with HCHS/SOL SNPs, because many of the reference individuals were genotyped with an Affymetrix array that had lower overlap with the HCHS/SOL Illumina array.

For local ancestry inference on the autosomes, we phased the HCHS/SOL data combined with the reference panels for the intersection SNPs using Beagle 4.0 (Browning and Browning 2007, https://faculty.washington.edu/browning/beagle/beagle.html). Although the HCHS/SOL data include individuals of known relationship, we did not use pedigree information in the phasing. We inferred local ancestry using RFMix (Maples *et al.* 2013, https://sites.google.com/site/rfmixlocalancestryinference/) version 1.5.4 with the PopPhased option and a minimum node size of 5, as recommended in the documentation. We did not use the RFMix EM option as it was not computationally feasible on a data set of this size.

RFMix does not provide a specific chromosome X setting. There are several factors to consider when choosing appropriate settings for X. Males are haploid for the nonpseudoautosomal regions of X, and thus their data on these regions of X has no phase uncertainty, whereas females are diploid for X and phase can be statistically inferred but not without error. It is common to code males as homozygous diploid on X; this is undesirable in the reference panel as it makes the observed male haplotypes look more frequent than they really are. In contrast, coding admixed males as homozygous should provide good results when using RFMix without the EM option because inference on one admixed haplotype does not affect inference on another admixed haplotype. In order to account for these factors with the available RFMix options, we artificially paired reference males into phased pseudodiploid individuals within each continental reference group, discarding the final male if the group had an odd number of males. RFMix does not attempt to rephase the reference individuals, so phase information is not lost by the pairing. We then either (Option 1) performed separate analyses for admixed males and admixed females, with admixed haploid males treated as perfectly phased and accounting for phase uncertainty in the admixed females, or (Option 2) analyzed all admixed individuals together, coding males as homozygous diploid and accounting for phase uncertainty in all the individuals. Option 1 allows analysis of the two sexes in parallel, which speeds computation, whereas Option 2 is easier to implement. We also investigated (Option 3) coding all males (reference and admixed) as homozygous. In Option 1 we used the TrioPhased option in RFMix to analyze the males, which makes use of the known phase of these individuals. For all other analyses we used the PopPhased option in RFMix, which accounts for phase uncertainty. Before running RFMix with any of these options, we phased chromosome X for all the admixed and reference individuals together using Beagle 4.0 (Browning and Browning 2007) with males coded as homozygous, which imputes missing data in the males as well as phasing the females.

It can be helpful to plot principal components (PCs) corresponding to the parts of individuals’ genomes that are derived from a specific continental ancestry (Moreno-Estrada *et al.* 2013). In order to do this, one can mask (set to missing) alleles corresponding to haplotypes inferred not to be of the specified ancestry. Because the missing pattern applies to haplotypes rather than genotypes, it is natural to apply PCs on a per-haplotype basis. Multidimensional scaling (MDS) with Euclidean distance produces PCs (Cox and Cox 1994; Gower 1966). We thus applied MDS with Euclidean distance (treating alleles as 0’s and 1’s) to the masked haplotype data using the dist() and cmdscale() functions in R (R Core Team, 2014), with masked positions treated as missing data. Missing data induced by the masking are accounted for when the distances between two haplotypes are calculated. Only positions at which both haplotypes are nonmissing are used in the distance calculation. If two haplotypes share no nonmasked positions, we set their distance to the mean distance between all pairs. Our MDS-based approach to ancestry-specific PCA is relatively straightforward. We provide an outline of the code used to generate results in this paper, including running RFMIX, masking haplotypes, and calculating ancestry-specific PCs (http://faculty.washington.edu/sguy/local_ancestry_pipeline/).

**Figure 1:**
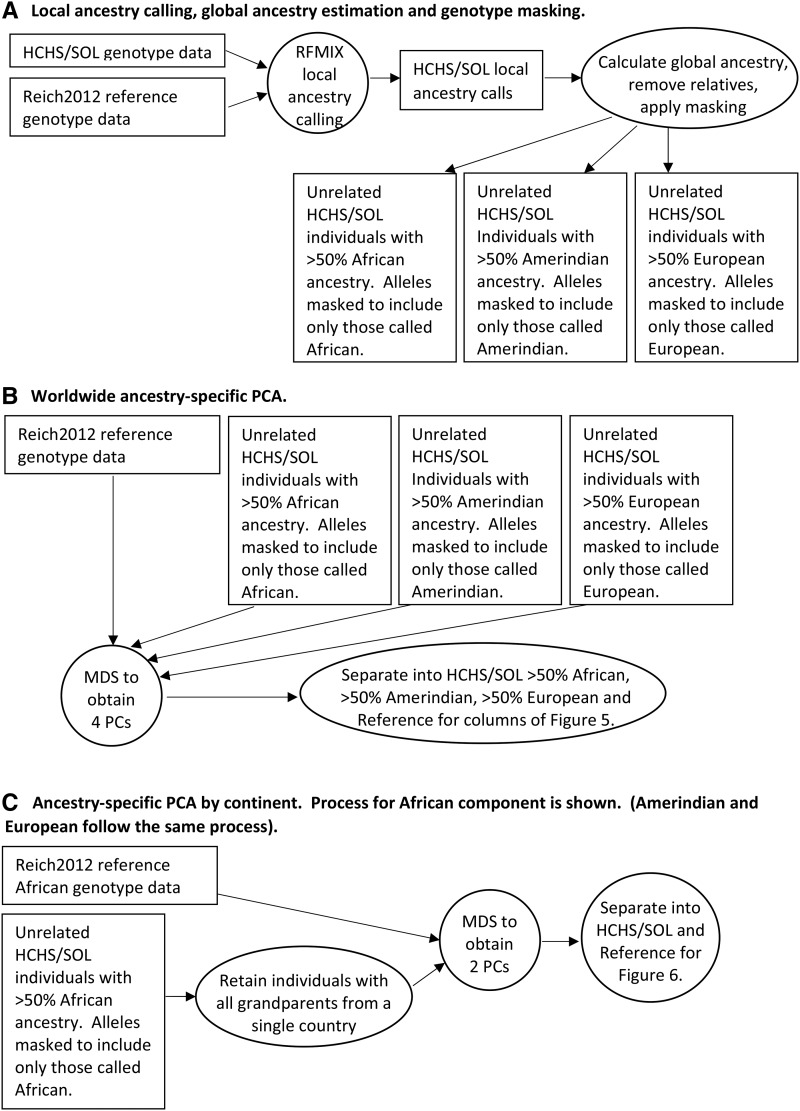
Processes used to obtain ancestry-specific PCA for Figure 5 and Figure 6. Data analyses and filtering are shown in circles/ovals, while data are shown in rectangles. HCHS/SOL, Hispanic Community Health Study/Study of Latinos; MDS, multidimensional scaling; PCA, PC analysis; PCs, principal components; Reich2012, reference panel derived from data published in Reich *et al.* (2012) combined with data from the 1000 Genomes Project.

We removed 2168 close relatives (Conomos *et al.* 2016) from the ancestry-specific PC analyses (Figure 1A). For a given continental ancestry, we used only individuals with at least 50% ancestry derived from that continent (calculated from the local ancestry calls), because individuals with low levels of ancestry will have little genetic data after masking and thus have noisy PC values. To avoid spurious PCs arising from genomic regions with high linkage disequilibrium, and to reduce computation time in calculating interhaplotype distances, we thinned markers to 10,000 SNPs across the autosomes.

We ran two sets of ancestry-specific PC analyses. We performed a single worldwide analysis that includes all continental ancestries (Figure 1B). The main purpose of this analysis is to check that the results from the local ancestry calling are reasonable. The African part of the admixed individuals’ genomes should cluster with the African reference individuals, for example. In order to investigate fine-scale structure more closely, we performed a second set of analyses comprised of three within-continent analyses (Figure 1C). These analyses use reference individuals from a single continental group (African, Amerindian, or European), along with the parts of the admixed individuals’ genomes that are called as having ancestry from that continental group. In this fine-scale analysis, we only include an admixed individual if his/her four grandparents are known to have come from the same country, and we label the individual by that country in the results.

### Data availability

The authors state that all data necessary for confirming the conclusions presented in the article are represented fully within the article.

## Results

Table 1 compares the autosomal calls from using the HGDP reference with those from using the Reich2012 reference. There is very little difference in the calls from the two analyses, with 98.7% of the calls being identical. Calls made from the HGDP reference are slightly more likely to be European and slightly less likely to be Amerindian than those from the Reich2012 reference, which may be due to the lower number of Amerindian reference individuals in the HGDP reference.

**Table 1:** Proportion of autosomal local ancestry calls

	Reich2012 African	Reich2012 Amerindian	Reich2012 European	Total
HGDP African	0.1388	0.0004	0.0023	0.1414
HGDP Amerindian	0.0003	0.3017	0.0029	0.3049
HGDP European	0.0023	0.0046	0.5468	0.5537
Total	0.1414	0.3067	0.5519	1.0000

At each SNP (single nucleotide polymorphism), each admixed individual has two called local ancestries for each reference panel. We match the two local ancestries obtained with each of the two reference panels so as to maximize the agreement. For example, if the call using the HGDP reference is African/European and the call using the Reich2012 reference is Amerindian/African, we assume that both calls are agreeing on one haplotype being of African descent, while disagreeing on whether the other haplotype is European or Amerindian. Reich 2012, reference panel derived from data published in Reich *et al.* (2012) combined with data from the 1000 Genomes Project; HGDP, reference panel derived from the Human Genome Diversity Project (Li *et al.* 2008) and The 1000 Genomes Project Consortium (2012).

We looked at Mendelian inconsistency rates (Baran *et al.* 2012) to compare the accuracy of the inferred local ancestry from analysis with the HGDP reference and the Reich2012 reference. Mendelian inconsistency rates have previously been used to compare methods (Baran *et al.* 2012; Pasaniuc *et al.* 2013) and to find regions of the genome with high rates of error in local ancestry calls (Pasaniuc *et al.* 2013). We say that a parent–offspring pair or mother–father–child trio are Mendelian inconsistent at a position if their ancestry calls at the locus are not consistent with the laws of Mendelian inheritance. For example, if a mother is called as having a chromosome of African origin and a chromosome of European origin at a locus, while the child is called as having two Amerindian chromosomes, then the inferred local ancestry is Mendelian inconsistent. Due to the household-based sampling design of the HCHS/SOL study, there are large numbers of parent–offspring pairs and trios. We analyzed 174 pairs and 203 trios. Table 2 shows Mendelian inconsistency rates for the autosomal data. The rates of Mendelian inconsistency are lower in pairs than in trios because errors in ancestry calls are more difficult to detect when one parent’s data are missing. Overall, the rates of inconsistency are low, and the rates are lower with calls from the HGDP reference than with calls from the Reich2012 reference.

**Table 2:** Mendelian inconsistency rates on the autosomes

	Pairs	Trios
HGDP reference	0.0009	0.0026
Reich2012 reference	0.0013	0.0034

Trios are mother–father–child trios, whereas pairs are mother–child or father–child with the other parent not being present in the data. HGDP, reference panel derived from the Human Genome Diversity Project (Li *et al.* 2008) and The 1000 Genomes Project Consortium (2012); Reich 2012, reference panel derived from data published in Reich *et al.* (2012) combined with data from the 1000 Genomes Project.

The main output from RFMix is most-likely ancestry calls, however, the program can also produce posterior probabilities that indicate how certain the algorithm is about its call (Maples *et al.* 2013). For each position and each haplotype in the individual there are three posterior probabilities for the three possible local ancestry calls. For example, the probabilities might be 0.99 for African, 0.001 for European, and 0.009 for Amerindian, indicating that the haplotype is almost certainly of African ancestry at this position. We recorded the highest posterior probability for each haplotype at each position (0.99 in the example), and report the autosomal averages in Table 3. Regardless of which ancestry has the highest posterior probability, the average highest posterior probability is close to 1, but is slightly higher for the HGDP reference than for the Reich2012 reference. Thus, both Mendelian inconsistency rates and average posterior probabilities indicate that the two reference sets are giving high accuracy but that the HGDP reference gives slightly better results. This is interesting as it indicates that, in this particular setting, the advantage of having a higher number of SNPs in the RFMix analysis (419,645 *vs.* 236,736 genome-wide) outweighs the disadvantage of having a reduced number of reference individuals (63 *vs.* 154 Amerindian reference individuals).

**Table 3:** Average highest posterior probabilities

	Overall	African	Amerindian	European
HGDP reference	0.9941	0.9918	0.9939	0.9948
Reich2012 reference	0.9932	0.9910	0.9931	0.9939

Average local ancestry posterior probability for the ancestry with the highest posterior probability at each position, reported by ancestry with the highest posterior probability and overall. HGDP, reference panel derived from the Human Genome Diversity Project (Li *et al.* 2008) and The 1000 Genomes Project Consortium (2012); Reich 2012, reference panel derived from data published in Reich *et al.* (2012) combined with data from the 1000 Genomes Project.

We used the HGDP reference in calling local ancestry on chromosome X. The Mendelian inconsistency rates for chromosome X in Table 4 demonstrate that Options 1 (separate analysis of haploid admixed males and diploid admixed females) and 2 (admixed males coded as homozygous) have similar accuracy, while Option 3 (admixed and reference males coded as homozygous) is only slightly inferior. The chromosome X mother–daughter pair rates (Table 4) are similar to the autosomal pair rates (Table 2), indicating that accuracy on X with these options is similar to autosomal accuracy. Local ancestry calls from Options 1 and 2 were 99.8% identical, while results from Option 3 were 99.6% identical to those from Options 1 or 2. We also calculated average highest posterior probabilities for the X chromosome. On this chromosome, results on males are not comparable across options due to the different diploid and haploid coding schemes, so we only present results from females (Table 5). Options 1 and 2 are almost identical in terms of average highest posterior probabilities for females, which is to be expected since these two options treat females identically. Option 3 has higher posterior probabilities, which may be because the RFMix program thinks it has a larger number of reference haplotypes due to duplication of male haplotypes and thus is, erroneously, more confident in its results. This illustrates one of the pitfalls of using posterior probabilities to assess accuracy, which is that they may be miscalibrated. We chose to proceed with Option 1 for future analyses of these data.

**Table 4:** Mendelian inconsistency rates on chromosome X

	Coding of Males in Admixed	Coding of Males in Reference	Mother–Daughter	Mother–Son	Mother–Father–Daughter
Option 1	Haploid	Haploid	0.0010	0.0017	0.0064
Option 2	Homozygous	Haploid	0.0011	0.0014	0.0063
Option 3	Homozygous	Homozygous	0.0012	0.0018	0.0074

Analyses were performed using the HGDP reference panel. See main text for further description of the analysis options. HGDP, reference panel derived from the Human Genome Diversity Project (Li *et al.* 2008) and The 1000 Genomes Project Consortium (2012).

**Table 5:** Average highest posterior probabilities in females on X

	Overall	African	Amerindian	European
Option 1	0.9929	0.9937	0.9927	0.9924
Option 2	0.9930	0.9937	0.9927	0.9925
Option 3	0.9931	0.9939	0.9927	0.9928

Average local ancestry posterior probability for the ancestry with the highest posterior probability at each position, reported by ancestry with the highest posterior probability and overall.

**Figure 2:**
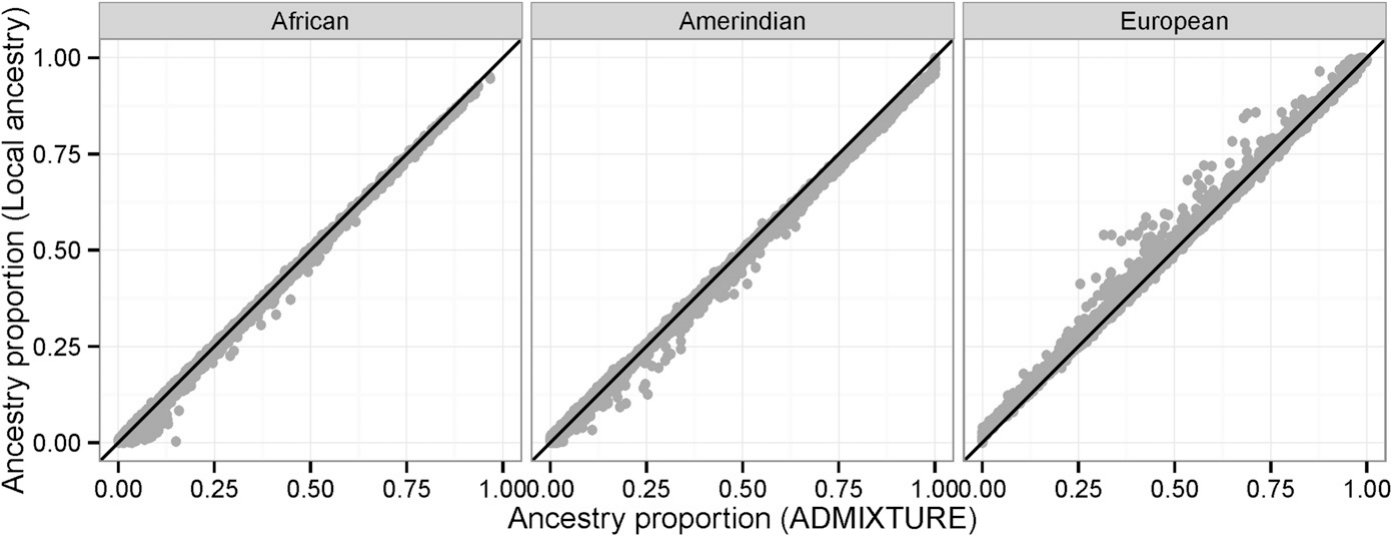
Autosome-wide average ancestry proportions from RFMIX calls (“local ancestry”) *vs.* ADMIXTURE.

Figure 2 shows average autosome-wide local ancestry call proportions for each unrelated admixed individual against global autosomal ancestry estimated using a supervised ADMIXTURE analysis (Alexander *et al.* 2009). ADMIXTURE was run with K = 3 populations, using reference individuals from the 1000 Genomes project and the HGDP. We see that RFMix is tending to call higher proportions of European ancestry and lower proportions of African and Amerindian ancestry. This may be because some of the African and Amerindian reference individuals have small amounts of European admixture, which biases the estimates of continental allele frequencies. As a result, ADMIXTURE may be assigning some of the European ancestry to the African and Amerindian components. RFMix and other local ancestry calling methods should be less susceptible to this issue because they look for long segments of continuous continental ancestry. Although a small portion of a European segment in an HCHS/SOL individual may match a haplotype seen in an Amerindian reference individual due to European ancestry in that individual, the RFMix algorithm will tend to call the segment as European because the overall evidence from the larger region is for European ancestry.

**Figure 3:**
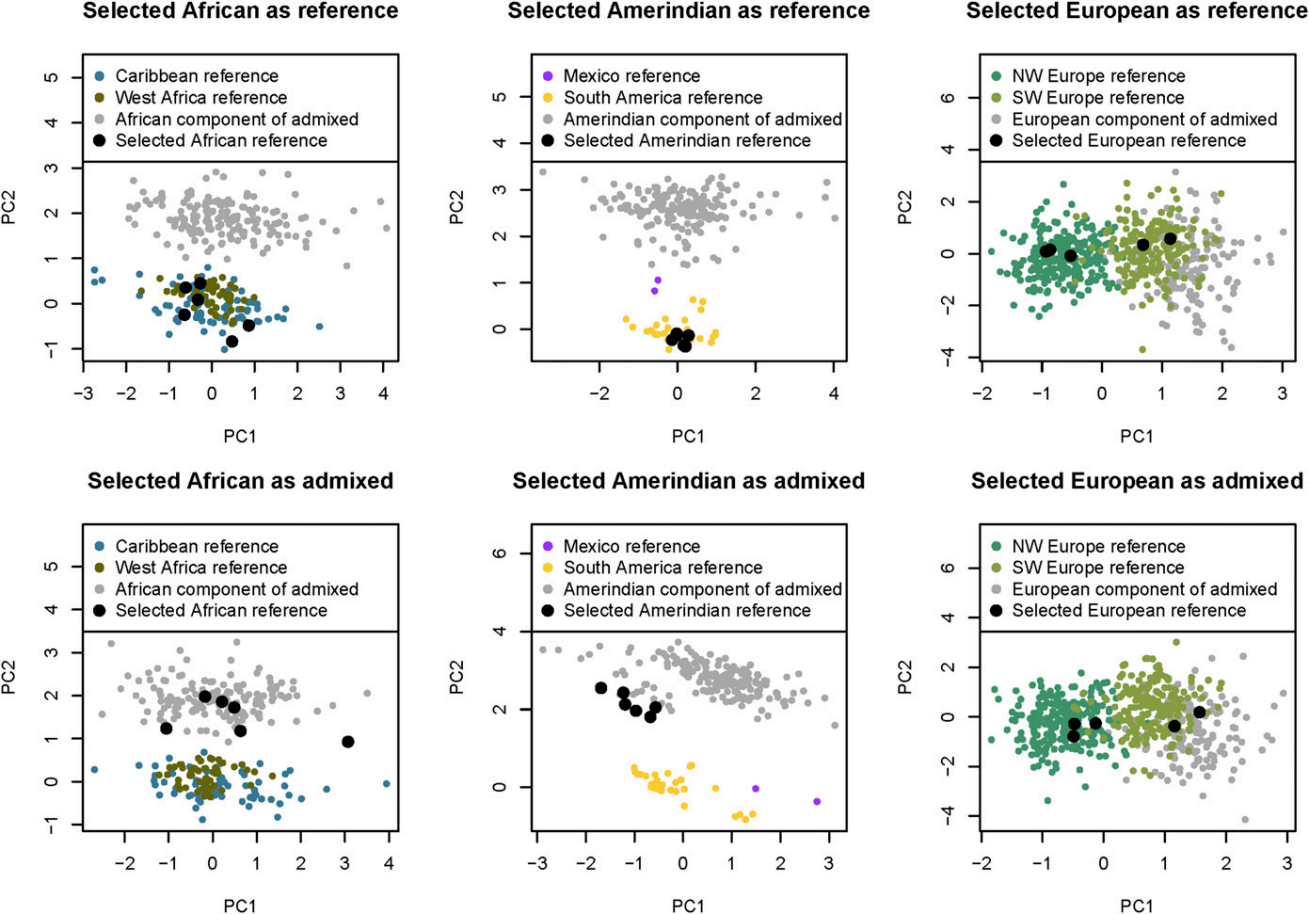
Example illustrating artifactual results from PCAmask. We performed separate analyses with PCAmask for each continent (column in the figure), including the appropriate HGDP reference individuals and 100 admixed individuals with at least 50% estimated ancestry from the specified continent. Alleles not estimated to be from the specified continent were masked out. The top row shows the original PCAmask analysis, while in the bottom row we reran PCAmask after moving three reference individuals (six haplotypes) from the reference file to the admixed file. The six selected reference haplotypes are shown as large black dots in both rows. PC, principal component.

A previous approach to obtaining ancestry-specific PCs was to compute the subspace spanned by the first k PCs by finding a matrix decomposition that minimizes the reconstruction error (Johnson *et al.* 2011; Moreno-Estrada *et al.* 2013). However, we found that when using the PCAmask program implementing this approach (https://sites.google.com/site/pcamask/home), we obtained artifactual separation of clusters between reference and admixed individuals. In order to investigate this behavior, for each continental ancestry, we ran PCAmask analyses using the HGDP reference data and genotypes of 100 unrelated HCHS/SOL individuals with at least 50% estimated ancestry from the specified continent, and masked genotypes at positions not called to be of the specified continental ancestry. In a second PCAmask analysis of the same individuals, we chose three reference individuals to move from the reference file to the admixed file, and added corresponding entries to the masking file (with no masking of these individuals). Thus, the input data of the two analyses was identical in content. Figure 3 shows that the ancestry-specific components of the admixed individuals cluster separately from the corresponding reference individuals in the PC plots, and that moving individuals from the reference file into the admixed file causes these individuals to move from the reference cluster to the admixed cluster, despite no change in the analyzed genotypes.

**Figure 4:**
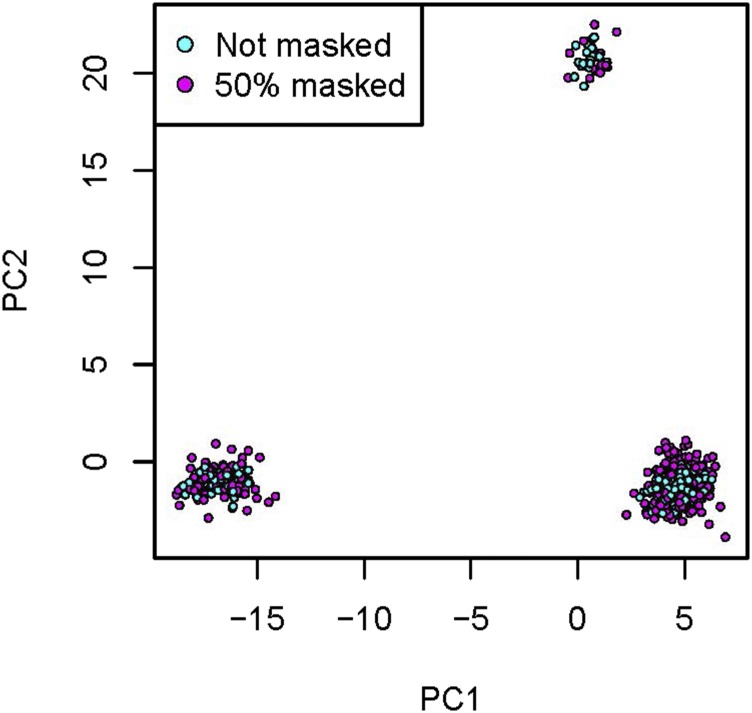
Effect of masking in the MDS-based PCA. We used MDS to calculate principal components for the reference individuals in the intersection of the Reich *et al.* (2012) and HGDP panels. Half the individuals (shown in cyan) had no missing data. In the other individuals (shown in magenta), we masked (set to missing) a random 50% of the alleles on each of the two haplotypes. HGDP, Human Genome Diversity Project; MDS, multidimensional scaling; PCA, PC analysis; PCs, principal components.

**Figure 5:**
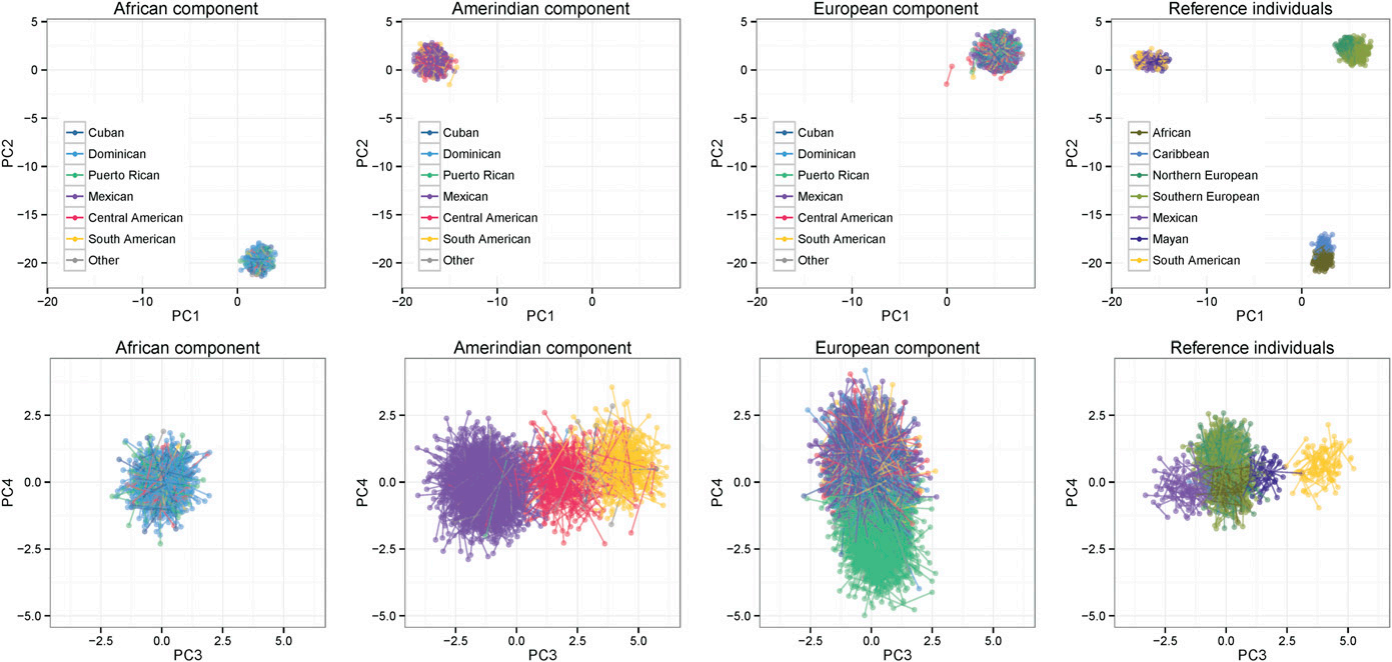
Worldwide ancestry-specific PCs. The top row shows PCs 1 and 2, while the bottom row shows PCs 3 and 4, as outlined in Figure 1B. The first three columns show the three ancestral components of the admixed individuals, while the fourth column shows the reference individuals. The values in the four columns were calculated together and may be compared, but are shown in separate plots for clarity. The two haplotypes from each individual are connected by a line. The legend in the first three columns indicates color-coding for the self-identified background groups of HCHS/SOL participants, while that in the fourth column indicates regional origin of reference individuals. HCHS/SOL, Hispanic Community Health Study/Study of Latinos; PCs, principal components.

**Figure 6:**
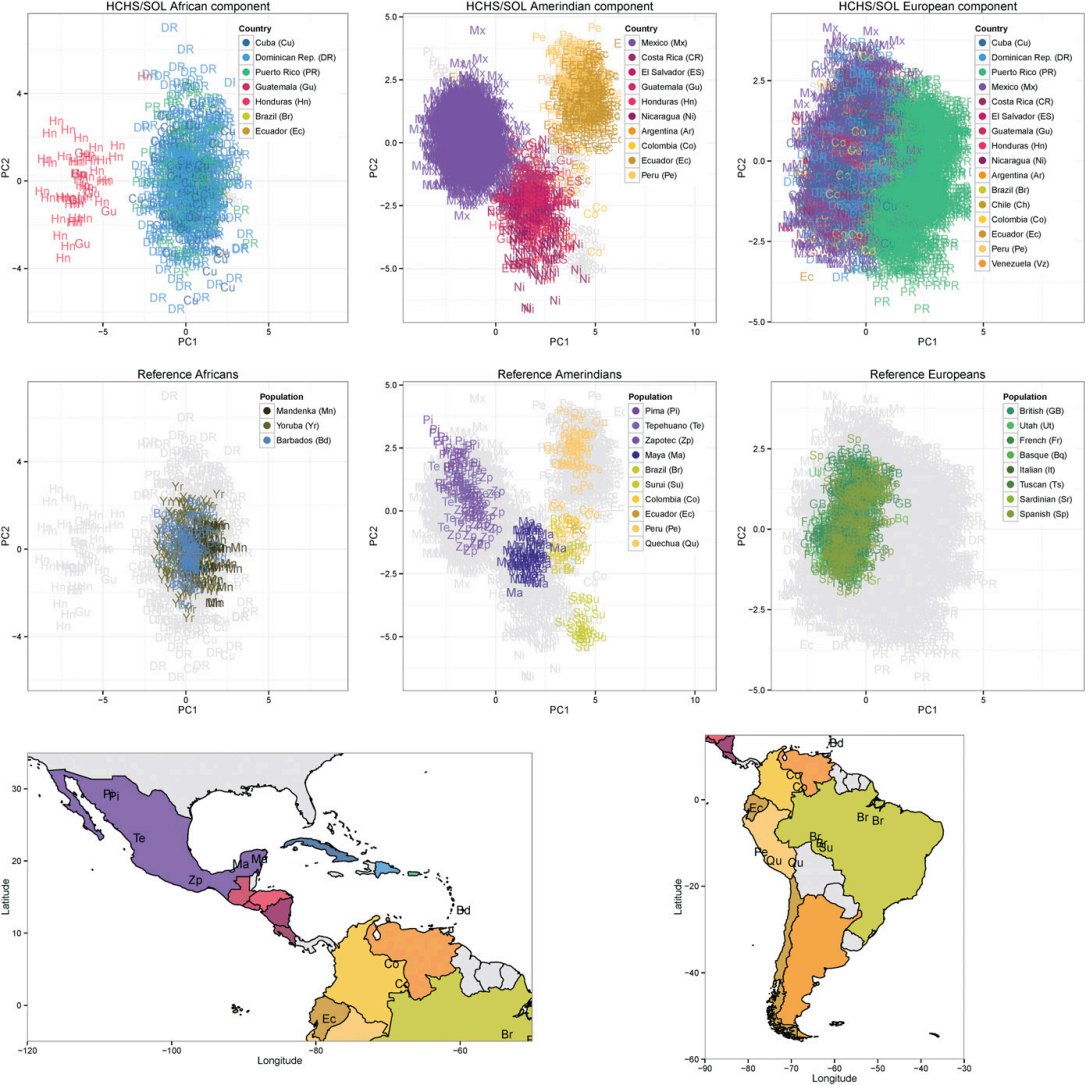
Ancestry-specific PCs for single ancestral continents. Each column of the top two rows of the figure clusters ancestry from one ancestral continental group (African, Amerindian, or European), with PCs calculated as indicated in Figure 1C. The top row highlights the HCHS/SOL individuals labeled by the country of origin of their grandparents (with reference individuals in the background in gray), while the middle row highlights the reference individuals (with HCHS/SOL individuals in the background in gray). The bottom row of the figure gives maps showing the locations of the populations. HCHS/SOL, Hispanic Community Health Study/Study of Latinos; PCs, principal components.

We investigated the effect of masking in our MDS approach by setting to missing a proportion of the genotype data in reference individuals. Figure 4 shows the result of masking 50% of the genotype data in selected individuals. These individuals cluster with the nonmasked individuals, demonstrating that the masking does not lead to artifactual cluster separation. Not surprisingly, because of the loss of information by masking, the masked individuals cluster slightly less tightly than the nonmasked individuals. Similarly, in the full data, we see that the ancestry-specific masked haplotypes of the admixed individuals are clustering with the corresponding reference haplotypes in all but a couple of cases, which are discussed in more detail below (see Figure 5 and Figure 6).

For the ancestry-specific PC analyses shown in Figure 5 and Figure 6, we used the reference individuals from the Reich2012 data set, because this data set covers a broader range of reference populations. We used the local ancestry calls derived from the RFMix run using the Reich2012 reference, which are almost identical to those from the RFMix run using the HGDP reference (Table 1). There were 513 unrelated admixed individuals with at least 50% African ancestry, 1997 with at least 50% Amerindian ancestry, and 5735 with at least 50% European ancestry.

In Figure 5, we plot ancestry-specific PCs from a worldwide analysis. The ancestry-specific parts of admixed individuals’ genomes cluster closely with the reference individuals from the same ancestry on PCs 1 and 2. The tight, well separated clusters for genomic segments identified with each of the three continental groups, and the lack of intermediates, indicates that the procedures used here are accurately delineating the boundaries of ancestral segments in multi-way admixed individuals. On PC 3, the Amerindian parts of admixed individuals’ genomes cluster with reference individuals from the American geographic region (Mexico, Central America, or South America) that matches the admixed individuals’ self-identified backgrounds. PC 4 shows that the European component partially differentiates individuals of Puerto Rican background from those of the other background groups. This PC 4 axis is not related to north–south geographic variation within the European reference population, and thus may primarily represent drift due to a founder effect.

To look more closely for within-continent ancestry structure, we calculated PCs for each continental ancestry separately, without the reference groups from the other two continents. We calculated four PCs per continental ancestry, but only show the first two in Figure 6 as the third and fourth components did not further illuminate population structure in the HCHS/SOL individuals.

The left panels of Figure 6 show PCs for African ancestry. The individuals from the Caribbean (primarily Dominican Republic and Cuba as country of grandparental origin) cluster with the reference Yoruba in Ibadan, Nigeria and the African Caribbeans in Barbados, but less so with the Mandenka. In contrast, the individuals from Central America (Honduras and Guatemala as country of grandparental origin) do not cluster closely with the African ancestry from the Caribbean individuals, or with the Yoruba or Mandenka. The variation is on the same axis (PC1) that separates Yoruba from Mandenka, so may represent an ancestry contribution from other populations within Africa. The populations of Honduras and Guatemala include the Garífuna people. Previous mitochondrial analysis of the Garífuna population suggests primarily West African ancestry, with possibly some Mozambiquan ancestry, and a population bottleneck (Salas *et al.* 2005). Thus, the separation of the Central American African component may be due to ancestry from African populations that are distinct from the Yoruba and Mandenka, or genetic drift due to a population bottleneck, or a combination of these two factors.

The center panels of Figure 6 show PCs for Amerindian ancestry. The individuals with grandparents from Central America tend to cluster with the reference group closest to Central America (the Maya). The individuals with grandparents from Mexico cluster with the other Mexican reference individuals (Pima, Tepehuano, and Zapotec) and the Maya. The individuals with grandparents from a South American country cluster with the South American reference individuals, with additional structure present. The individuals with grandparents from Peru cluster with the reference individuals from Peru and the Quechua individuals. The individuals with grandparents from Colombia cluster with the reference individuals from Colombia and Brazil. The individuals with grandparents from Ecuador tend to cluster with the Peruvian and Colombian reference individuals. Previous studies have also found population structure within the Amerindian ancestral component of admixed individuals from the Caribbean, Mexico, and South America (Moreno-Estrada *et al.* 2014, 2013; Gravel *et al.* 2013; Homburger *et al.* 2015).

The right panels of Figure 6 show PCs for European ancestry. As in Figure 5, Puerto Rican European ancestry diverges from that of the other admixed groups, and does so on an axis of variation (here PC1) that does not reflect the north–south geographical gradient in the reference European samples.

## Discussion

We used RFMix to infer three-way (African, European, and Amerindian) ancestry in the HCHS/SOL individuals. Our local ancestry calls are being used for admixture mapping with the HCHS/SOL data (Schick *et al.* 2016). We will also use the local ancestry calls to calculate ancestry-specific allele frequency estimates, which will be used to investigate the ancestral origins of variants associated with disease-related phenotypes in the HCHS/SOL data in future work.

The unprecedented size of our study, with over 12,000 admixed Hispanic/Latino individuals, allowed us to stress-test RFMix and to investigate within-ancestry population structure in finer detail than previous studies. Due to the large numbers of parent–offspring relationships in our sample, we were able to calculate Mendelian inconsistency rates, which allowed us to compare approaches and to confirm that RFMix is giving consistent results.

Due to the colonial history of the Americas, it is challenging to obtain reference individuals of nonadmixed Amerindian ancestry. We compared two reference panels, one with more markers but fewer Amerindian reference individuals, and the other with more Amerindian reference individuals but fewer markers overlapping with those in the HCHS/SOL data. We found that the former gave slightly superior results in our data, as measured by Mendelian inconsistency rates and posterior local ancestry probabilities. Further studies will be needed to see the extent to which this result generalizes to other data sets and to the use of other local ancestry calling methods. Computational constraints prevented us from using RFMix’s EM option, which augments the reference panel with the admixed individuals after an initial round of local ancestry calling; the use of an EM-type option in other studies might further favor higher marker density over higher numbers of reference individuals.

Like many genetics software tools, RFMix was designed only for autosomal data. We investigated three approaches to analyzing the X chromosome with RFMix. We assessed the results using Mendelian inconsistency rates. The two best approaches involved pairing haploid males into pseudodiploid individuals in the reference panel in order to avoid the double-counting that would occur if these individuals were coded as homozygous diploid. However, coding reference males as homozygous resulted in only a small loss of accuracy.

We calculated global ancestry proportions from the autosomal local ancestry calls, and compared these to global ancestry calls obtained directly from the genotypes using ADMIXTURE. We found that the ADMIXTURE results tended to have lower proportions of European ancestry and higher proportions of African and Amerindian ancestry. It seems likely that local ancestry calls, which are based on large chromosomal segments rather than individuals’ SNPs, are less susceptible to bias due to low levels of admixture in the reference individuals. Thus, global ancestry proportions based on local ancestry calls are likely to be superior to direct estimates of global ancestry using methods such as ADMIXTURE.

Ancestry-specific PCA, calculated using inferred local ancestry, can be used to examine the fine-scale structure of the data without confounding by differing ancestry proportions between individuals. For example, in the usual ‘global’ PCA of autosomal SNP genotypes, we found a cluster of HCHS/SOL individuals with high African ancestry and Central American background, which separated from individuals with high African ancestry and Caribbean background [the small yellow cluster in the lower left corner of Figure 3a of Conomos *et al.* (2016)]. In this previous global analysis, it was not clear whether the Central American cluster separated from the Caribbean cluster because of their different Amerindian ancestries, or because their African origins are different. The local ancestry analysis presented here strongly favors the latter interpretation. Similarly, the global PCA showed that individuals of Puerto Rican background are well-separated from other background groups. The continental ancestry-specific analysis presented here suggests that differences in European ancestry, likely due to a founder effect, contribute to this separation, which was not apparent from the global analysis.

We first attempted to use PCAmask (Moreno-Estrada *et al.* 2013) to perform ancestry-specific PCA, but found that it produced artifactual separation of reference and ancestry-specific admixed individuals. Moreno-Estrada *et al.* (2013) infer divergence between the European ancestry component of Caribbean individuals and current day Iberian individuals based on results from PCAmask. Our study shows that separation between reference and admixed individuals in results from PCAmask can be artifactual. We then developed a straight-forward approach for ancestry-specific PCA using existing statistical software tools. Based on the results obtained on our data, this approach does not seem to be susceptible to artifactual separation of reference and admixed individuals.

Our ancestry-specific PC analysis of the HCHS/SOL data revealed clear population structure within each continental ancestry across the individuals in the HCHS/SOL study. These results provide support for the HCHS/SOL analysis plan to separate individuals into genetic analysis groups (Cuban, Dominican, Puerto Rican, Mexican, Central, and South American) based on self-identified background and PCA, and to use these groups in association analyses (Conomos *et al.* 2016). Due to the within-continental structure, some causal genetic variants may be specific to a certain subgroup, and appropriate use of the group variables may increase power to find associations with such variants. Furthermore, allele frequencies calculated within ancestral genomic segments can identify the origins of risk alleles and guide the selection of replication cohorts. In interpreting the results of our analyses, it is important to note that the study individuals came from just four centers within the US (Chicago, IL; Miami, FL; Bronx, NY; San Diego, CA), and are thus not necessarily representative of the US Hispanic/Latino population as a whole.
